# Prevalence and correlates of suicide attempts in high-risk populations: a cross-sectional study among patients receiving opioid agonist therapy in Norway

**DOI:** 10.1186/s12888-022-03829-y

**Published:** 2022-03-15

**Authors:** Jørn Henrik Vold, Else-Marie Løberg, Christer F. Aas, Jan Alexander Steier, Kjell Arne Johansson, Lars Thore Fadnes

**Affiliations:** 1 Department of Addiction Medicine, Haukeland University Hospital, Bergen, Norway; 2 Department of Global Public Health and Primary Care, University of Bergen, Bergen, Norway; 3 Department of Psychiatry, Haukeland University Hospital, Bergen, Norway; 4 Department of Clinical Psychology, University of Bergen, Bergen, Norway

**Keywords:** Substance-related disorders, Suicide attempts, Opiate substitution treatment, Injecting substance use

## Abstract

**Background:**

Death by suicide in patients enrolled in opioid agonist therapy (OAT) is a major clinical concern. However, little knowledge exists regarding suicide attempts in this patient group. This study presents the lifetime prevalence of suicide attempts and the associations between suicide attempts and clinical and sociodemographic variables such as education, sex, early onset of substance use (< 13 years of age), substance use patterns, and injecting substance use among patients receiving OAT.

**Methods:**

We used data from a cohort of OAT patients in Norway obtained from a health assessment of self-reported suicide attempts and sociodemographic and clinical factors. A total of 595 patients receiving OAT were assessed from 2016 to 2020. A binary logistic regression analysis was performed and reported with an unadjusted odds ratio and 95% confidence intervals (OR). The purpose of this assessment was to analyze associations between suicide attempts and substance use patterns as well as the injection of substances during the 30 days leading up to the health assessment. A negative binomial regression analysis with an incidence rate ratio and 95% confidence intervals (IRR) was performed to investigate sex, education, early onset of substance use, and the number of suicide attempts.

**Results:**

Forty-one percent of the OAT patients had attempted to die by suicide at least once during their lifetime. An early onset of substance use was strongly associated with the suicide attempts (IRR: 1.7, 1.3–2.2). No significant association was found between suicide attempts and sex (IRR: 1.2, 0.9–1.6) or education (IRR: 0.6, 0.2–2.1). Likewise, no association was identified between suicide attempts and injecting substance use (OR: 0.9, 0.6–1.3), nor using alcohol (OR: 0.9, 0.7–1.3), amphetamines (OR: 1.0, 0.7–1.3), benzodiazepines (OR: 1.0, 0.7–1.4), cannabis (OR: 1.2, 0.9–1.7), cocaine (OR: 1.3, 0.6–3.0), or opioids (OR: 1.4, 0.9–2.0).

**Conclusion:**

The lifetime prevalence of suicide attempts was alarmingly high in the OAT population. An early onset of substance use seemed to be an important risk factor for suicide attempts. There was a non-significant association to more current use of opioids among OAT patients with previous suicide attempts.

**Supplementary Information:**

The online version contains supplementary material available at 10.1186/s12888-022-03829-y.

## Background

Nearly two out of five patients entering substance use disorder (SUD) treatment have attempted suicide during their lifetime [1–3]. Suicide attempts among SUD patients are, in part, attributable to a chaotic life situation consisting of unstable housing and living conditions, hard substance use, violence, physical and mental traumatic events, and comorbid mental health disorders [4, 5]. In 2020, the European Union’s Drug Strategy for 2020–2025 aimed to improve personal mental health and reduce premature deaths in European countries for marginalized SUD patients [6]. As a part of this strategy, the European Union called for better characterization of mental health in various SUD populations in order to reduce barriers to treatment and facilitate targeted treatment. Suicide attempts may lead to suicide, and they are a major clinical concern in this patient population [7]. The literature is scarce on the prevalence of suicide attempts among various SUD populations, and there is a substantial lack of significance attributed to associated risk factors [8].

Opioid agonist therapy (OAT) is a well-known treatment approach used by SUD patients to recover from opioid dependence [9]. Although OAT may improve mental health symptoms [10, 11] and reduce opioid-related deaths and illegal opioid use [12–15], OAT patients usually have mental disorder comorbidities strongly associated with suicide [16]. Suicide is the second leading limiting factor in OAT patient prognosis; overdose-related accidents are the most common cause [17]. Globally, the estimates of suicide attempts among heroin users vary substantially up to 47 % [18–20], while one study among those entering OAT estimated that nearly one out of five had attempted suicide ever [16]. Furthermore, heroin users who inject substances are up to 14 times more likely to die from suicide than the general population [21]. This corresponds to a suicide lifetime prevalence from 3-35 % among this population [22].

Patients receiving OAT usually have a history of hard injecting opioid use, often initiated in adolescence or early adulthood [23]. Additionally, polysubstance use, including substances used alongside OAT opioids, is common among OAT patients, with a prevalence of up to 70 % in this population [24, 25]. While OAT may protect against suicide [9, 10], polysubstance use is often a substantial contributor to reduced quality of life, homelessness, greater psychological distress, poorer social functioning, and mental disorder comorbidities – such as major depressive symptoms and borderline personality disorder [24, 26–31]. This places OAT patients at a high lifetime risk of attempting suicide [22]. However, little is known about the impact of early onset of substance use, types of substances used, injecting substance use, and level of education on previous suicide attempts among OAT patients [32, 33].

The primary objective of the present study is to estimate the lifetime prevalence of suicide attempts among patients with opioid dependence who received opioid agonist therapy (OAT). The secondary objective is to investigate the association between suicide attempts and other factors. More specifically, we will:

1. estimate the lifetime prevalence of suicide attempts
2. investigate the associations between sex, level of education, early onset of substance use, and suicide attempts.
3. evaluate the associations between previous suicide attempts and current injecting substance use and use of alcohol, amphetamines, benzodiazepines, cannabis, cocaine, and opioids.

## Methods

### Data source

We used data from a cohort nested in the INTRO-HCV study in Bergen and Stavanger, Norway [34]. Data were collected from May 2016 to July 2020, and patients were recruited from OAT outpatient clinics in the aforementioned cities. All patients received OAT, meaning they met the criteria for opioid dependence syndrome according to the international statistical classification of diseases and related health problems, version 10 (ICD-10), and received OAT medication daily prior to and during the study period.

### Data collections

During a health assessment, the included patients were assessed regarding their suicide attempts, current substance use and injecting substance use, and other sociodemographic and clinical data. The data were stored in a health register using electronic data collection software (Checkware) under the supervision of trained research nurses. The clinical data––including the level of education, severe infectious diseases (hepatitis C virus, hepatitis B virus, and human immunodeficiency virus infections), suicide attempts, injecting substance use, and substance use––were collected from their electronic medical record. A total of 595 OAT patients were recruited during the study period. The mean time that patients had spent in OAT was eight years with a standard deviation (SD) of five years. The study sample was substantially similar to the national OAT population and the OAT populations in Sweden and the UK concerning sex, type of OAT medication used, and mean age [31, 35, 36]. However, there was a higher prevalence of substance use, particularly amphetamines, in the 30 days leading up to the health assessment compared with the national OAT assessment (Table 1) [37].

**Table 1:** Basic characteristics of 595 opioid agonist therapy patients (numbers (n) and percentages (%)):

	All patients (*N* = 595)
Age (years), n (%)	
18-30	57 (10)
30-40	169 (28)
40-50	193 (32)
50-60	10 (23)
≥ 60	36 (6)
Mean (SD)	44 (10)
Sex, n (%)	
Male	420 (71)
Female	175 (29)
Highest level of education, n (%)	
Not completed primary school	30 (5)
Completed primary school (9 years)	266 (45)
Completed high school (12 years)	243 (41)
≤ 3 years of college or university	46 (8)
> 3 years of college or university	10 (2)
Number of years in opioid agonist therapy, mean (SD)	8 (5)
Injecting substance use past 30 days, n (%)	181 (30)
Unstable housing past 30 days^a)^, n (%)	58 (10)
Substances used past 30 days^b)^, n (%)	
Cannabis	396 (67)
Benzodiazepines	354 (60)
Alcohol	349 (59)
Amphetamines	249 (42)
Opioids	123 (21)
Cocaine	25 (4)
Mean age for the onset of substance use (mean (SD))	13 (2)
Comorbidities, n (%)	
Hepatitis C virus infection	350 (59)
Hepatitis B virus infection	< 5 (< 1)
Human immunodeficiency virus	< 5 (< 1)

*SD* Standard deviation

^a^An unstable housing was defined as living in a homeless shelter or with family or friends at any time during the 30 days leading up to the health assessment. Having owned or rented housing situation or being imprisoned were defined as a stable housing

^b^The number of patients who have used substances at least once during the 30 days leading up to the health assessment

### Definition of study variables

A suicide attempt was defined as any self-reported attempted suicide during a lifetime. The suicide variable was divided into five groups: zero, one, two, three, or four or more suicide attempts. We defined ‘receiving OAT’ according to the OAT medication (buprenorphine or methadone) stated during the health assessment. The level of education was defined as the highest level of education completed, and this was categorized into five classes: “not completed primary school”, “completed primary school (nine years)”, “completed high school (12 years)”, “completed three years or less of college or university”, and “completed more than three years of college or university.” We set “injecting substance use” as having injected at least once during the 30 days leading up to the health assessment. Similarly, substance use was divided into six classes: “alcohol,” “amphetamines,” “benzodiazepines,” “cannabis,” “cocaine,” and “opioids,” according to the use during the past 30 days. The early onset of substance use was defined categorically as those using any kinds of substances––including amphetamines, alcohol, benzodiazepines, cannabis, cocaine, or opioids––before the age of 13 (< 13). In the absence of a definition of early onset of substance use in the literature, thirteen years of age was set as a cut-off, referring to the mean age for the onset of substance use in the study sample and the point when entering the lower secondary school, often associated with being first-time offered substances [38]. Moreover, we assessed the extent of severe infectious diseases as markers of the study sample’s comorbidities. This was done by drawing blood samples to test for chronic infectious diseases during the health assessment. Current infections were defined as detecting hepatitis C virus RNA by polymerase chain reaction (hepatitis C virus infection), hepatitis B virus surface antigens (hepatitis B virus infection), or human immunodeficiency virus antigens/antibodies (human immunodeficiency virus infection). Blood samples were analyzed at the Department of Laboratory Medicine at Haukeland University Hospital in Bergen, Norway, and the Department of Medical Biochemistry and Microbiology at Stavanger University Hospital in Stavanger, Norway (accredited by ISO standard 15189).

### Statistical analyses

We used Stata/SE 17.0 (StataCorp, TX, USA) for the descriptive and regression model analyses. Microsoft Excel was used to create a bar chart and IBM SPSS version 26.0 was used to expectation-maximization imputation. Unless otherwise stated, the threshold for statistical significance was set to *P* < 0.05 for all analyses.

We dealt with any missing values concerning the potential suicide attempt risk factors–– including the variables of types of substances used, early onset of substance use, and injecting substance use––as ‘missing at random’ when running expectation-maximization imputation. We identified missing values in 10 % in these factors and all were replaced with estimated values by imputation.

We performed a logistic regression analysis reported with an odds ratio and 95 % confidence intervals (OR) to investigate the unadjusted association between suicide attempts [binary exposure variable (yes/no)] and injecting substance use, as well as the use of amphetamines, benzodiazepines, cocaine, cannabis, and opioids [binary outcome variables (yes/no)]. Furthermore, a negative binomial regression analysis reported with an incidence rate ratio and 95 % confidence intervals (IRR) was performed to investigate the unadjusted association between sex, level of education, and the early onset of substance use (categorical exposure variables) and the number of suicide attempts (numeric outcome variable). If the number of suicide attempts exceeded four, it was handled as four suicide attempts in the analysis. As a sensitivity analysis, we performed a similar negative binomial regression analysis without categorizing the number of suicide attempts into classes in the outcome variable. Moreover, as a robustness check, we run a binary logistic regression to assess the association between early onset of substance use [binary exposure variable (yes/no)] and suicide attempt [binary outcome variables (yes/no)]. The Stata commands for the analyses are presented in Additional File 1.

### Ethics approval and consent to participate

The study was reviewed and approved by the Regional Ethical Committee for Health Research West, Norway (REK Vest 2017/51). Each patient provided written informed consent prior to enrolling in the study.

## Results

### Patients’ characteristics

A total of 71% of the study sample participants were males, and the mean age was 44 years (SD: 10 years). Five percent of the participants had not completed primary school (nine years), and 45% had primary school (nine years) listed as their highest level of education. Seventy-nine percent had consumed at least one substance, and 30% had injected substances during the 30 days leading up to the health assessment. The mean onset age for substance use was 13 years (SD: 2 years).

### Lifetime prevalence of suicide attempts

**Fig. 1:**
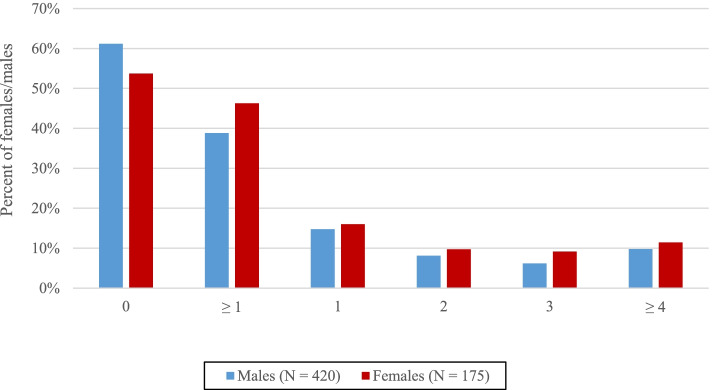
Title: The number of suicide attempts among patients receiving OAT. Legends: OAT: Opioid agonist therapy. The figure displays the number of OAT patients who have attempted zero, one, two, three, and four or more suicide attempts during their lifetime

A total of 41% of the study sample participants had attempted suicide at least once (Fig. 1), with prevalence estimates of 46% among females and 39% among males. Additionally, 10% reported four or more suicide attempts during their lifetime.

Unadjusted associations between suicide attempts and substance use, as well as injecting substance use

No association was found between suicide attempts (as an exposure factor) and injecting substance use (OR: 0.9, 0.6–1.3), as well as using substances: alcohol (OR: 0.9, 0.7–1.3), amphetamines (OR: 1.0, 0.7–1.3), benzodiazepines (OR: 1.0, 0.7–1.4), cannabis (OR: 1.2, 0.9–1.7), cocaine (OR: 1.3, 0.6–3.0), or opioids (OR: 1.4, 0.9–2.0) (Table 2). There was a non-significant trend towards using opioids, cannabis, and cocaine among patients attempting suicide. Similar results were seen using the non-imputed data (Additional File 2).

**Table 2:** Binary logistic regression of the association between previous suicide attempts and current substance use and injecting use (*N* = 595)

	**Unadjusted OR (95 % CI)**						
	Injecting substance use during the past 30 days	Substance use during the past 30 days					
		Alcohol	Amphetamines	Benzodiazepines	Cannabis	Cocaine	Opioids
Number of patients^a)^	72	140	101	146	169	12	58
Suicide attempt (ever)	0.9 (0.7–1.3)	0.9 (0.7–1.3)	1.0 (0.7–1.3)	1.0 (0.7–1.4)	1.2 (0.9–1.7)	1.3 (0.6–3.0)	1.4 (0.9–2.0)

*OR* Odds ratio, *CI* Confidence Interval

Legends: The table displays the unadjusted OR of the association between suicide attempts, injecting substance use, and the types of substances used during the 30 days leading up to the health assessment

^a^Number of patients who attempted suicide ever and used substances or injected substances during the 30 days leading up to the health assessment

Unadjusted associations between early onset of substance use, level of education, sex, and suicide attempts

The onset of substance use under 13 years of age (as an exposure factor) was strongly associated with suicide attempts (IRR: 1.7, 1.3–2.2) (Table 3). However, no significant association was found between sex (as an exposure factor) and suicide attempts (IRR: 1.2, 0.9–1.6). Likewise, not completing primary school and completing primary school (nine years) (IRR: 0.8, 0.4–1.5), high school (IRR: 0.8, 0.4–1.5), three years or less of college or university (IRR: 1.0, 0.5–2.1), or more than three years of college or university (IRR: 0.6, 0.2–2.1) (as exposure factors) were not associated with suicide attempts. Similar results were found using the non-imputed data (Additional File 3), binary logistic regression analysis of the association between early onset of substance use and previous suicide attempts, and not categorizing the number of suicide attempts into classes (outcome variable) in the negative binomial regression analysis (Additional File 4).

**Table 3:** Negative binomial regression of the association between suicide attempts and their risk factors (*N* = 595)

	Number of patients^1)^	IRR (95 % CI)
		Suicide attempt
Sex		
Male	163	1.0 (ref.)
Female	81	1.2 (0.9–1.6)
Level of education		
- Not completed primary school	15	1.0 (ref.)
- Completed primary school (9 years)	109	0.8 (0.4–1.5)
- Completed high school (12 years)	98	0.8 (0.4–1.5)
- ≤ 3 years of college or university	19	1.0 (0.5–2.1)
- > 3 years of college or university	< 5	0.6 (0.2–2.1)
Early onset of substance use^2)+3)^	137	1.7 (1.3–2.2)

*IRR* Incidence rate ratio, *CI* Confidence interval

1) Number of patients who attemped suicide (ever) (244 patients), stratified on sex, level of education, and early onset of substance use

2) The early onset of substance use is defined as substance use before the age of 13 (< 13).

3) As a robustness check, the assocation between the early onset of substance use [binary exposure variables (yes/no)] and suicide attempts (ever) [binary outcome variables (yes/no)] was assessed using a logistic regression analysis. The result showed OR 1.7, 95 % CI: 1.2 - 2.3

Legends: The table displays the association between the level of education, sex, early onset of substance use, and suicide attempts among patients who received opioid agonist therapy

## Discussion

The present study showed that two out of five patients had attempted to die by suicide during their lifetime; females tended to have slightly higher rates than males. Being introduced to substances before the age of 13 was strongly associated with suicide attempts when compared to being introduced after this age. Nevertheless, completing primary school (nine years) or higher education was not associated with suicide attempts when compared to non-completion. Suicide attempts were not significantly associated with the current use of substances or the injection of substances. However, there was a non-significant trend towards using opioids, cannabis, and cocaine among patients attempting suicide.

The lifetime prevalence of suicide attempts in the general population is around 4 % [39], substantially lower than observed in this high-risk OAT population. Although OAT may protect against suicide [9], the present suicide attempt prevalence is unfortunately in line with or slightly exceeds the suicide attempt prevalence of several severe mental disorders, such as bipolar disorder (34%) [40], schizophrenia (25%–50%) [41, 42], and borderline personality disorder (60%–70%) [43]. Furthermore, the impact of comorbid mental disorders on suicide attempts among OAT patients was significant [44, 45], suggesting an even higher risk of suicide attempts for OAT patients with comorbid mental disorders than those without mental comorbidities [46–49]. In Norway, where 15% of OAT patients report current depressive symptoms and 7% have delusions [37], these underlying mental symptoms are likely associated with the high prevalence of suicide attempts in this population [9].

The early onset of substance use, in the present study––meaning substance use before reaching the age of 13––was strongly associated with suicide attempts in the OAT population. Although little is known about the early onset of substance use and suicide attempts among OAT patients, the results align with several studies investigating suicide attempts among adolescents with SUD [50, 51]. Being exposed to substance use at an early age is usually a significant risk factor for hard injecting substance use and relapsing after SUD treatment [52–54]. Additionally, patients with early initiation of substance use are more likely to become pregnant earlier, become unemployed, and commit crimes [55–57]. Furthermore, among adolescents with SUD attempting suicide, early onset of mental disorders, particularly mood disorders, disruptive behavior disorder, and attention deficit-hyperactivity disorder, are common [50], with some sex differences. Among males with hard substance use in adolescence, suicide attempts and co-occurring problem behavior are frequently reported. In contrast, early onset of smoking or alcohol use in adolescence is associated with suicide attempts among females. Thus, among OAT patients, where hard substance use is common during adolescence or early adulthood [23], several of these medical and psychosocial risk factors for suicide attempts are often presented. This might be a significant reason for the high prevalence of suicide attempts among OAT patients and should lead to close clinical monitoring for suicide risk in this population.

The current types of substances used were not associated with suicide attempts. This adds essential information to the existing literature in this population. Although substance use and previous suicide attempts are individual predictors for suicide [58], these results showed that the current types of substances used were insignificant for OAT patients’ high prevalence of suicide attempts. On the other hand, if a positive association between the types of substances used and suicide attempts was revealed, this could be an additional risk factor for suicide attempts to keep in mind when managing OAT patients regarded as being at risk of suicide.

To our knowledge, no studies have evaluated suicide attempt associations with the current types of substances used alongside OAT medication. Although no association was found, polysubstance use is a significant risk factor for suicide found in global suicide predictions among SUD patients [59]. While OAT may reduce suicide rates among patients with opioid dependence [9], the increasing number of opioid users without OAT––following the current opioid epidemic in the US––is a likely cause of the increasing suicide rates seen over the past decade [60, 61]. This may be attributable in part to more comorbidities in the population, of which polysubstance use––particularly opioids and benzodiazepines––is an essential contributor [62, 63]. However, in the Norwegian OAT population, where polysubstance use is high and remains substantially unchanged over time, the incidence rate of premature deaths–– including suicide, injuries, and murder––has remained steady in recent years [37]. This may support our findings, particularly those showing no associations between suicide attempts and the type of current substances used.

### Strengths and limitations

A major strength of this study is its relatively large sample size of 595 patients receiving OAT who are typically difficult to reach in health-care. However, one important limitation of this study is the data structure with few data points: this limits possible assessment of factors, as they can only be assumed to be either prior to or after suicide attempts. This made it difficult to make a causal inference model. Thus, we opted to present crude associations for some key factors where we had underlying hypotheses. To some degree, this may also reduce the generalizability of the results to other settings or groups that do not receive OAT. Another limitation of this study can be found in the self-reported data aspect. The individuals’ definition of suicide attempts may vary significantly, and their abilities to remember suicide attempts introduce recall bias. Similarly, recognizing the consumption of different types of substances and their frequency of use might be difficult.

## Conclusion

Forty-one percent of patients receiving OAT had attempted suicide at least once in their lives. Early onset of substance use was associated with suicide attempts. There was a non-significant association with more current use of opioids among patients with previous suicide attempts. In this population, there is an urgent need for further understanding of the issues concerning suicide behavior and prevention.

## Supplementary Information

**Additional file 1.**

**Additional file 2.**

**Additional file 3.**

**Additional file 4.**

## Data Availability

The datasets analyzed during the current study are not publicly available due data protection requirements but are available from the corresponding author on reasonable request.

## Abbreviations

ICD-10: The international statistical classification of diseases and related health problems, version 10
IRR: Incidence rate risk
OAT: Opioid agonist therapy
OR: Odds ratio
SD: Standard deviation
SUD: Substance use disorder
