# Supplementary material for: Prevalence and correlates of suicide attempts in high-risk populations: a cross-sectional study among patients receiving opioid agonist therapy in Norway
Source: BMC Psychiatry. 2022 Mar 15;22:181. doi: 10.1186/s12888-022-03829-y (PMC8922793; doi:10.1186/s12888-022-03829-y)
Supplement: Supplementary file 1 — Additional file 1. [file 12888_2022_3829_MOESM1_ESM.docx]

Additional File 1

Unadjusted logistic regression analysis of the association between suicide attempts (ever) and current substance use and injecting substance use:

Alcohol: logistic alcohol i.suicideattempt

Amphetamines: logistic amphetamines i.suicideattempt

Benzodiazepines: logistic benzodiazepines i.suicideattempt

Cannabis: logistic cannabis i.suicideattempt

Cocaine: logistic cocaine i.suicideattempt

Opioids : logistic opioids i.suicideattempt

Unadjusted negative binomial regression analyses of the association between suicide attempts and their risk factors:

Sex: nbreg suicideattempts i.sex,irr

Level of education: nbreg suicideattempts i.education,irr

Early onset of substance use: nbreg suicideattempts i.earlyonset,irr

A robustness check of the early onset of substance use [binary exposure variables (yes/no)] and suicide attempts (ever) [binary outcome variables (yes/no)] using a logistic regression analysis (referred in Table 2 (footnote)):

logistic suicideattempt i.earlyonset
